# Crystal structures and Hirshfeld analysis of 4,6-di­bromo­indole­nine and its quaternized salt

**DOI:** 10.1107/S2056989021011385

**Published:** 2021-11-02

**Authors:** Irina S. Konovalova, Svitlana V. Shishkina, Dmytro Kobzev, Olha Semenova, Anatoliy Tatarets

**Affiliations:** a State Scientific Institution Institute for Single Crystals of the National Academy of Sciences of Ukraine, 61001, Kharkov, Ukraine; b Department of Chemical Sciences, Ariel University, Ariel, 40700, Israel

**Keywords:** crystal structure, Hirshfeld surface, 4,6-di­bromo-2,3,3-trimethyl-3*H*-indole, quaternized salt

## Abstract

4,6-Di­bromo-2,3,3-trimethyl-3*H*-indole, C_11_H_11_Br_2_N, exists as a neutral mol­ecule in the asymmetric unit. The asymmetric unit of 4,6-di­bromo-2,3,3-trimethyl-3*H*-indol-1-ium iodide, C_12_H_14_Br_2_N^+^·I^−^, contains one organic cation and one iodine anion. The positive charge is localized on the quaternized nitro­gen atom. In the crystal, mol­ecules of 4,6-di­bromo­indole­nine are linked by C—Br⋯π halogen bonds, forming zigzag chains propagating in the [001] direction. The mol­ecules of the salt form layers parallel to the (010) plane where they are linked by C—H⋯Br hydrogen bonds, C—Br⋯Br and C—Br⋯I halogen bonds. The Hirshfeld surface analysis and two dimensional fingerprint plots were used to analyse the inter­molecular contacts present in both crystals.

## Abstract 2

In the crystal, mol­ecules of 4,6-di­bromo­indole­nine are linked by C—Br⋯π halogen bonds, forming zigzag chains propagating in the [001] direction. The mol­ecules of the salt form layers parallel to the (010) plane where they are linked by C—H⋯Br hydrogen bonds C—Br⋯Br and C—Br⋯I halogen bonds.

## Chemical context

The structural analysis of 2,3,3-trimethyl-3*H*-indole (2,3,3-tri­methyl­indole­nine) and its quaternized salts (Lynch *et al.*, 2012 ▸; Connell *et al.*, 2014 ▸) plays a crucial role in understanding the mechanisms of the chemical reactions resulting in various functional products. These inter­mediates are promising scaffolds for the synthesis of indole­nine-containing fluorescent dyes, including highly versatile cyanine (Sun *et al.*, 2016 ▸; Feng *et al.*, 2020 ▸) and squaraine dyes (Beverina & Salice, 2010 ▸). The incorporation of heavy atoms in the mol­ecule, such as bromine and iodine, increases the generation of reactive species during photosensitization (Szaciłowski *et al.*, 2005 ▸; Semenova *et al.*, 2021 ▸). In particular, fluorescent dyes with bromine atoms are utilized for photodynamic therapy applications (Atchison *et al.*, 2017 ▸; Liu *et al.*, 2021 ▸). Moreover, cyanine with the 4,6-di­bromo­indole­nine moiety indicates excellent properties for optical tumor imaging by its fluorescence (Guerrero *et al.*, 2017 ▸). 
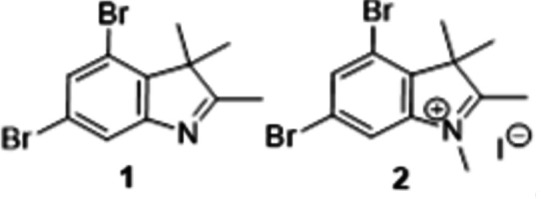


**Figure 1:**
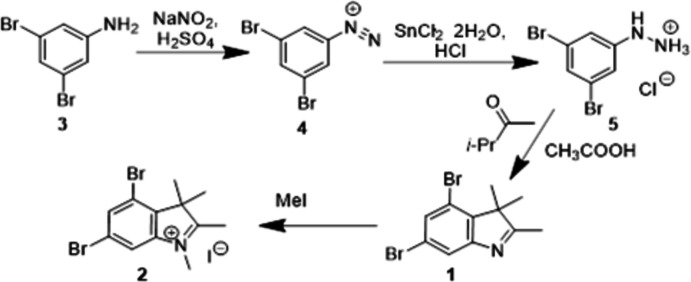
Synthesis of the title compounds **1** and **2**.

In this work, we carried out an X-ray diffraction and Hirshfeld surface analysis of 4,6-di­bromo­indole­nine (**1**) and its quaternized salt (**2**), crystals of which were obtained by sequential synthesis starting from 3,5-di­bromo­aniline (**3**) by its diazo­tization with nitro­sylsulfuric acid in sulfuric acid followed by reduction of the diazo­nium salt **4** with tin(II) chloride. The resulting 3,5-di­bromo­phenyl­hydrazine was refluxed with 3-methyl-2-butanone in acetic acid to give 4,6-di­bromo­indole­nine, **1**, which after *N*-alkyl­ation with the excess of iodo­methane in benzene solution forms crystals of the quaternized indolium salt **2** (Fig. 1 ▸).

## Structural commentary

**Figure 2:**
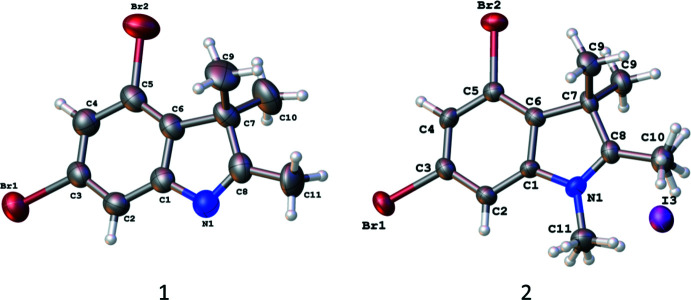
Mol­ecular structure of compounds **1** and **2** with the atom labelling. Displacement ellipsoids are drawn at the 50% probability level.

In the crystal, 4,6-di­bromo-2,3,3-trimethyl-3*H*-indole, **1**, exists as one neutral mol­ecule in the asymmetric unit (Fig. 2 ▸). The quaternized mol­ecule **2** exists as a salt with an iodine anion in the crystal phase (Fig. 2 ▸). All atoms of the quaternized cation, with exception of the C9 atom and the hydrogen atoms of the C10H_3_ and C11H_3_ methyl groups are located in a special position relative to the symmetry plane. In compound **2**, the positive charge is localized on the nitro­gen atom, which is caused by its quaternization. The N1—C11 bond is shortened to 1.460 (10) Å in comparison with the mean value of 1.485 Å for an N—C*sp* ^3^ bond (Burgi & Dunitz, 1994 ▸). An analysis of the bond lengths in both structures showed that they are typical of those in similar compounds (Seiler *et al.*, 2018 ▸; Connell *et al.*, 2014 ▸; Holliman *et al.*, 2009 ▸; Bellêtete *et al.*, 1993 ▸).

## Supra­molecular features

**Figure 3:**
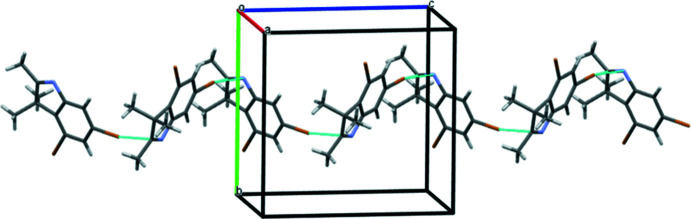
Zigzag chains in the crystal of compound **1**.

**Figure 4:**
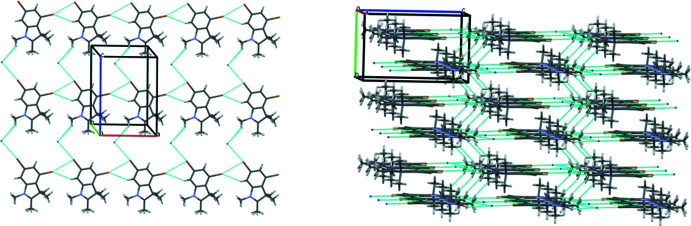
The chains (left) and layers (right) in the crystal of compound **2**.

In the crystal, mol­ecules of **1** form zigzag chains in the [001] direction as a result of the formation of inter­molecular C3—Br1⋯N1(π) and C3—Br1⋯C8(π) halogen bonds (Table 1 ▸, Fig. 3 ▸). Neighbouring chains are linked by weak C11—H11*C*⋯N1 hydrogen bonds (Table 1 ▸). It should be noted that only one of the bromine atoms participates in these inter­actions. The presence of the iodide anion in compound **2** leads to the complete involvement of both bromine atoms in the formation of inter­molecular inter­actions. As a result, mol­ecules of **2** form chains in the [100] direction as a result of the C2—H2⋯Br2 hydrogen bond and C5—Br2⋯Br1 halogen bond (Table 2 ▸, Fig. 4 ▸). Neighbouring chains are connected through the bridged iodide anion by the strong C3—Br1⋯I1 halogen bond and C11—H⋯I1 hydrogen bond. Layers parallel to the (010) plane can be recognized as a structural motif in the structure of **2** (Table 2 ▸).

## Hirshfeld surface analysis

**Figure 5:**
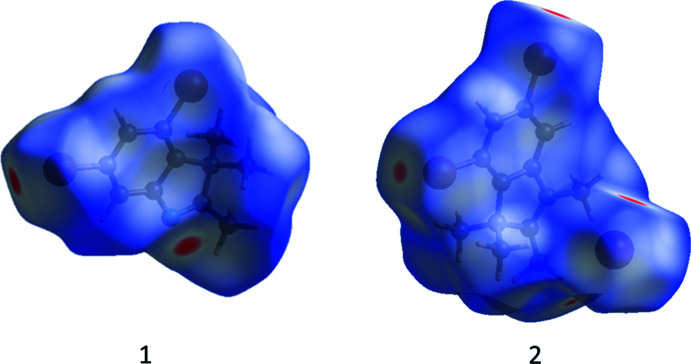
The Hirshfeld surface of compounds **1** and **2** mapped over *d*_norm_.

**Figure 6:**
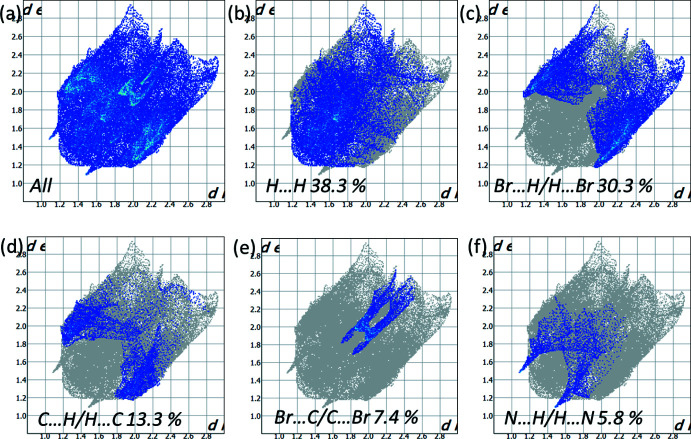
(*a*) The two-dimensional fingerprint plot for compound **1**, and those delineated into (*b*) H⋯H (38.3%), (*c*) Br⋯H/H⋯Br (30.3%), (*d*) C⋯H/H⋯C (13.3%), (*e*) Br⋯C/C⋯Br (7.4%) and (*f*) N⋯H/H⋯N (5.8%) contacts.

**Figure 7:**
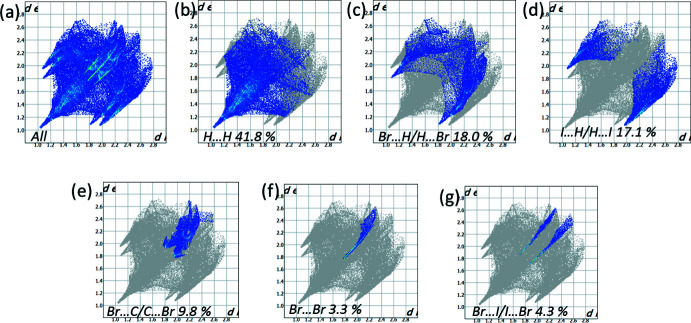
(*a*) The two-dimensional fingerprint plot for compound **2**, and those delineated into (*b*) H⋯H (41.8%), (*c*) Br⋯H/H⋯Br (18.0%), (*d*) I⋯H/H⋯I (17.1%), (*e*) Br⋯C/C⋯Br (9.8%) (*f*) Br⋯I/I⋯Br (4.3%) and Br⋯Br (3.3%) contacts.

*Crystal Explorer* 17.5 (Turner *et al.*, 2017 ▸) was used to analyse the inter­actions in the structures and fingerprint plots mapped over *d*_norm_ (Figs. 5 ▸–7 ▸ ▸) were generated. The mol­ecular Hirshfeld surfaces were obtained using a standard (high) surface resolution with the three-dimensional *d*_norm_ surfaces mapped over a fixed colour scale of −0.1256 (red) to 1.401 (blue). The areas in red on the *d*_norm_-mapped Hirshfeld surfaces (Fig. 5 ▸) correspond to contacts that are shorter than van der Waals radii sum of the closest atoms. As can be seen in Fig. 5 ▸, short contacts in **1** are present at the nitro­gen and Br1 atoms. In **2**, the areas of short contacts are located at both the bromine atoms, the iodine atom and the hydrogen atoms of the methyl groups (Fig. 5 ▸). All of the inter­molecular inter­actions of the title compounds are shown in the two-dimensional fingerprint plot presented in Figs. 6 ▸ and 7 ▸. The contribution of the Br⋯H/H⋯Br contacts, corresponding to the C—H⋯Br inter­action, is represented by a pair of sharp spikes. The inter­actions appear in the middle of the scattered points in the two-dimensional fingerprint plot with a contribution to the overall Hirshfeld surface of 30.3% (Fig. 6 ▸ *c*) and 18.0% (Fig. 7 ▸ *c*). The fingerprint plots indicate that the principal contributions are from H⋯H (38.3% (Fig. 6 ▸ *b*) in **1**; 41.8% (Fig. 7 ▸ *b*) in **2**), C⋯H/H⋯C (13.3%; Fig. 6 ▸ *d* in structure **1**) and I⋯H/H⋯I (17.1%; Fig. 7 ▸ *d* in structure **2**) contacts. The fingerprint plots also indicate that all inter­molecular inter­actions in the title compounds are rather weak.

## Database survey

A search of the Cambridge Structural Database (CSD, Version 5.42, update of November 2020; Groom *et al.*, 2016 ▸) for the 2,3,3-trimethyl-3*H*-indole skeleton yielded 306 hits. The most similar to the title compounds are 5,7-di­bromo-2,3,3-trimethyl-3*H*-indole (CSD refcode KOFRII; Holliman *et al.*, 2009 ▸) and 1,2,3,3-tetra­methyl-3H-indolium iodide (NENZAJ01; Connell *et al.*, 2014 ▸). These compounds have a very similar molecular structure and differ only in the position of the substituents.

## Synthesis and crystallization

**Synthesis of 4,6-di­bromo-2,3,3-trimethyl-3** ***H*** **-indole (1)**

3,5-Di­bromo­phen­yl)hydrazine hydro­chloride (**5**) (3.3 g, 11 mmol) and 3-methyl-2-butanone (1.8 mL, 16.8 mmol) were refluxed in 15 mL of acetic acid for 5 h. The acetic acid was evaporated and the residue was washed with a 5% aqueous solution Na_2_CO_3_ (20 mL) and then with water. Indole **1** was extracted using 3 × 25 mL of diethyl ether. The combined organic layers were dried over Na_2_SO_4_ and the ether was removed under reduced pressure by a rotary evaporator. After recrystallization from aceto­nitrile, light-brown crystals were obtained. Yield: 1.95 g (57%), ^1^H NMR (400 MHz, DMSO-*d*_6_), δ, ppm: 7.66 (1H, *s*, CH), 7.58 (1H, *s*, CH), 2.24 (3H, *s*, CH_3_), 1.36 [6H, *s*, (CH_3_)_2_]. Analysis, %: found C, 41.69; H, 3.49; N, 4.45, C_11_H_11_Br_2_N requires C, 41.67; H, 3.50; N, 4.42, ESI–MS *m*/*z* found: [*M* + H]^+^ 317.9; C_11_H_12_Br_2_N^+^ requires 317.9.

**Synthesis of 4,6-di­bromo-1,2,3,3-tetra­methyl-3H-indol-1-ium iodide (2)**

4,6-Di­bromo-2,3,3-trimethyl-3*H*-indole (**1**) (0.3 g, 0.95 mmol) was dissolved in benzene (5 mL), iodo­methane was added (0.5 mL, 8.03 mmol) and the mixture was left at room temperature for 24 h in a sealed tube. The beige crystals that formed were filtered off, washed with diethyl ether, dried, and were used without further purification. Yield: 300 mg (69%), ^1^H NMR (400 MHz, DMSO-*d*_6_), δ, ppm: 8.34 (1H, *s*, CH), 8.11 (1H, *s*, CH), 3.94 (3H, *s*, CH_3_), 2.81 (3H, *s*, CH_3_), 1.63 [6H, *s*, (CH_3_)_2_]. Analysis, %: found C, 31.34; H, 3.01; N, 3.08, C_12_H_14_Br_2_IN requires C, 31.40; H, 3.07; N, 3.05, ESI–MS *m*/*z* found: [*M* − I]^+^ 331.9; C_12_H_14_Br_2_N^+^ requires 332.0.

## Refinement

Crystal data, data collection and structure refinement details are summarized in Table 3 ▸. H atoms were included in calculated positions and treated as riding on their parent C atom: C—H = 0.93–0.98 Å with *U*_iso_(H) = 1.5U_eq_(C-meth­yl) or 1.2*U*_eq_(C) for all other H atoms.

## Supplementary Material

Crystal structure: contains datablock(s) 1, 2. DOI: 10.1107/S2056989021011385/zn2011sup1.cif

Structure factors: contains datablock(s) 1. DOI: 10.1107/S2056989021011385/zn20111sup4.hkl

Structure factors: contains datablock(s) 2. DOI: 10.1107/S2056989021011385/zn20112sup5.hkl

Supporting information file. DOI: 10.1107/S2056989021011385/zn20111sup4.cml

Supporting information file. DOI: 10.1107/S2056989021011385/zn20112sup5.cml

CCDC references: 2118325, 2118324

Additional supporting information:  crystallographic information; 3D view; checkCIF report

## Figures and Tables

**Table 1:** Hydrogen-bond geometry (Å, °) for **1** [Chem scheme1]

*D*—H⋯*A*	*D*—H	H⋯*A*	*D*⋯*A*	*D*—H⋯*A*
C3—Br1⋯N1^i^	1.89 (1)	3.19	5.283 (1)	166
C3—Br1⋯C8^i^	1.89 (1)	3.53	5.046 (1)	153
C11—H11*C*⋯N1^ii^	0.96	2.69	3.621 (1)	164

Symmetry codes: (i) -x+{\script{1\over 2}}, -y+1, z+{\script{1\over 2}}; (ii) x+{\script{1\over 2}}, -y+{\script{3\over 2}}, -z+1.

**Table 2:** Hydrogen-bond geometry (Å, °) for **2** [Chem scheme1]

*D*—H⋯*A*	*D*—H	H⋯*A*	*D*⋯*A*	*D*—H⋯*A*
C2—H2⋯Br2^i^	0.93	3.05	3.872 (1)	149
C5—Br2⋯Br1^ii^	1.88 (1)	3.58	5.397 (1)	162
C3—Br1⋯I1^iii^	1.90 (1)	3.62	5.514 (1)	176
C11—H11*A*⋯I1^iv^	0.96	3.14	3.881 (1)	135

Symmetry codes: (i) x-1, y, z; (ii) x+1, y, z; (iii) -x+1, -y+1, -z+1; (iv) -x+1, -y+1, -z.

**Table 3:** Experimental details

	**1**	**2**
Crystal data		
Chemical formula	C_11_H_11_Br_2_N	C_12_H_14_Br_2_N^+^·I^−^
*M*_r_	317.03	458.96
Crystal system, space group	Orthorhombic, *P*2_1_2_1_2_1_	Monoclinic, *P*2_1_/*m*
Temperature (K)	293	293
*a*, *b*, *c* (Å)	8.7761 (5), 11.3876 (7), 11.8654 (4)	8.3507 (6), 7.3719 (5), 11.7180 (8)
α, β, γ (°)	90, 90, 90	90, 92.755 (6), 90
*V* (Å^3^)	1185.81 (11)	720.53 (9)
*Z*	4	2
Radiation type	Mo *K*α	Mo *K*α
μ (mm^−1^)	6.80	7.74
Crystal size (mm)	0.4 × 0.3 × 0.3	0.4 × 0.2 × 0.1

Data collection		
Diffractometer	Xcalibur, Sapphire3	Xcalibur, Sapphire3
Absorption correction	Multi-scan (*CrysAlis PRO*; Rigaku OD, 2018 ▸)	Multi-scan (*CrysAlis PRO*; Rigaku OD, 2018 ▸)
*T*_min_, *T*_max_	0.682, 1.000	0.355, 1.000
No. of measured, independent and observed [*I* > 2σ(*I*)] reflections	8516, 2086, 1789	4499, 1374, 1242
*R*_int_	0.081	0.083
(sin θ/λ)_max_ (Å^−1^)	0.595	0.595

Refinement		
*R*[*F* ^2^ > 2σ(*F* ^2^)], *wR*(*F* ^2^), *S*	0.047, 0.121, 1.07	0.046, 0.121, 1.05
No. of reflections	2086	1374
No. of parameters	130	97
H-atom treatment	H-atom parameters constrained	H-atom parameters constrained
Δρ_max_, Δρ_min_ (e Å^−3^)	0.44, −0.55	0.86, −0.91
Absolute structure	Flack *x* determined using 613 quotients [(*I* ^+^)−(*I* ^−^)]/[(*I* ^+^)+(*I* ^−^)] (Parsons et al., 2013 ▸)	–
Absolute structure parameter	0.05 (2)	–

Computer programs: *CrysAlis PRO* (Rigaku OD, 2018 ▸), *SHELXT2014/5* (Sheldrick, 2015*a* ▸), *SHELXL2016/6* (Sheldrick, 2015*b* ▸) and *OLEX2* (Dolomanov *et al.*, 2009 ▸).

## supplementary crystallographic information

### 4,6-Dibromo-2,3,3-trimethyl-3H-indole (1) Crystal data

**Table:**

C_11_H_11_Br_2_N	*D*_x_ = 1.776 Mg m^−^^3^
*M**_r_* = 317.03	Mo *K*α radiation, λ = 0.71073 Å
Orthorhombic, *P*2_1_2_1_2_1_	Cell parameters from 2796 reflections
*a* = 8.7761 (5) Å	θ = 3.6–24.9°
*b* = 11.3876 (7) Å	µ = 6.80 mm^−^^1^
*c* = 11.8654 (4) Å	*T* = 293 K
*V* = 1185.81 (11) Å^3^	Prism, red
*Z* = 4	0.4 × 0.3 × 0.3 mm
*F*(000) = 616	

### 4,6-Dibromo-2,3,3-trimethyl-3H-indole (1) Data collection

**Table:**

Xcalibur, Sapphire3 diffractometer	2086 independent reflections
Radiation source: fine-focus sealed X-ray tube, Enhance (Mo) X-ray Source	1789 reflections with *I* > 2σ(*I*)
Graphite monochromator	*R*_int_ = 0.081
Detector resolution: 16.1827 pixels mm^-1^	θ_max_ = 25.0°, θ_min_ = 2.9°
ω scans	*h* = −10→10
Absorption correction: multi-scan (CrysAlisPro; Rigaku OD, 2018)	*k* = −13→13
*T*_min_ = 0.682, *T*_max_ = 1.000	*l* = −14→14
8516 measured reflections	

### 4,6-Dibromo-2,3,3-trimethyl-3H-indole (1) Refinement

**Table:**

Refinement on *F*^2^	Hydrogen site location: inferred from neighbouring sites
Least-squares matrix: full	H-atom parameters constrained
*R*[*F*^2^ > 2σ(*F*^2^)] = 0.047	*w* = 1/[σ^2^(*F*_o_^2^) + (0.0554*P*)^2^ + 0.295*P*] where *P* = (*F*_o_^2^ + 2*F*_c_^2^)/3
*wR*(*F*^2^) = 0.121	(Δ/σ)_max_ < 0.001
*S* = 1.07	Δρ_max_ = 0.44 e Å^−^^3^
2086 reflections	Δρ_min_ = −0.55 e Å^−^^3^
130 parameters	Absolute structure: Flack *x* determined using 613 quotients [(*I*^+^)-(*I*^-^)]/[(*I*^+^)+(*I*^-^)] (Parsons et al., 2013)
0 restraints	Absolute structure parameter: 0.05 (2)
Primary atom site location: dual	

### 4,6-Dibromo-2,3,3-trimethyl-3H-indole (1) Special details

**Table:**

Geometry. All esds (except the esd in the dihedral angle between two l.s. planes) are estimated using the full covariance matrix. The cell esds are taken into account individually in the estimation of esds in distances, angles and torsion angles; correlations between esds in cell parameters are only used when they are defined by crystal symmetry. An approximate (isotropic) treatment of cell esds is used for estimating esds involving l.s. planes.

### 4,6-Dibromo-2,3,3-trimethyl-3H-indole (1) Fractional atomic coordinates and isotropic or equivalent isotropic displacement parameters (Å^2^)

**Table:**

	*x*	*y*	*z*	*U*_iso_*/*U*_eq_	
Br1	0.24163 (11)	0.35819 (9)	0.83683 (7)	0.0630 (4)	
Br2	0.73559 (14)	0.21032 (9)	0.56768 (8)	0.0769 (4)	
N1	0.5082 (8)	0.6304 (7)	0.5315 (6)	0.0518 (18)	
C1	0.4908 (9)	0.5231 (8)	0.5910 (6)	0.0432 (19)	
C2	0.3837 (10)	0.5003 (7)	0.6734 (6)	0.047 (2)	
H2	0.312700	0.556729	0.694752	0.056*	
C3	0.3861 (9)	0.3912 (8)	0.7227 (6)	0.046 (2)	
C4	0.4903 (9)	0.3063 (8)	0.6936 (7)	0.052 (2)	
H4	0.489872	0.233692	0.729463	0.062*	
C5	0.5979 (10)	0.3311 (8)	0.6085 (7)	0.049 (2)	
C6	0.5999 (9)	0.4389 (8)	0.5579 (6)	0.046 (2)	
C7	0.6962 (9)	0.4972 (8)	0.4670 (6)	0.050 (2)	
C8	0.6202 (9)	0.6146 (8)	0.4635 (7)	0.051 (2)	
C9	0.6811 (15)	0.4323 (11)	0.3518 (7)	0.077 (3)	
H9A	0.719284	0.353684	0.359071	0.116*	
H9B	0.738795	0.473434	0.295515	0.116*	
H9C	0.575831	0.429848	0.329832	0.116*	
C10	0.8638 (11)	0.5078 (11)	0.5014 (10)	0.079 (3)	
H10A	0.870442	0.542173	0.575093	0.119*	
H10B	0.916473	0.556660	0.448193	0.119*	
H10C	0.909473	0.431228	0.502393	0.119*	
C11	0.6680 (12)	0.7123 (9)	0.3846 (8)	0.072 (3)	
H11A	0.597667	0.776545	0.390964	0.107*	
H11B	0.668219	0.683767	0.308475	0.107*	
H11C	0.768433	0.738629	0.404338	0.107*	

### 4,6-Dibromo-2,3,3-trimethyl-3H-indole (1) Atomic displacement parameters (Å^2^)

**Table:**

	*U*^11^	*U*^22^	*U*^33^	*U*^12^	*U*^13^	*U*^23^
Br1	0.0625 (6)	0.0687 (6)	0.0578 (5)	−0.0033 (6)	0.0196 (5)	0.0087 (4)
Br2	0.0836 (7)	0.0721 (7)	0.0750 (6)	0.0279 (7)	0.0213 (6)	0.0029 (5)
N1	0.054 (4)	0.052 (4)	0.050 (4)	−0.005 (4)	0.004 (3)	0.000 (4)
C1	0.042 (4)	0.048 (5)	0.040 (4)	−0.006 (4)	−0.002 (3)	−0.005 (4)
C2	0.045 (5)	0.048 (5)	0.047 (4)	0.002 (4)	0.003 (4)	−0.004 (4)
C3	0.039 (4)	0.052 (5)	0.045 (4)	−0.007 (4)	0.004 (4)	−0.004 (4)
C4	0.055 (5)	0.054 (6)	0.046 (4)	0.000 (5)	0.000 (4)	0.008 (4)
C5	0.043 (4)	0.063 (6)	0.041 (4)	−0.001 (4)	0.003 (4)	−0.001 (4)
C6	0.043 (5)	0.058 (5)	0.038 (4)	−0.003 (4)	0.005 (4)	0.001 (4)
C7	0.044 (5)	0.065 (6)	0.040 (4)	0.004 (4)	0.009 (3)	0.004 (4)
C8	0.045 (5)	0.063 (6)	0.047 (5)	−0.014 (4)	0.000 (4)	0.002 (4)
C9	0.095 (8)	0.092 (8)	0.046 (5)	−0.002 (7)	0.020 (5)	−0.016 (5)
C10	0.042 (6)	0.105 (10)	0.090 (7)	−0.003 (6)	0.008 (5)	0.013 (7)
C11	0.080 (7)	0.071 (7)	0.063 (5)	−0.019 (6)	0.023 (5)	0.020 (5)

### 4,6-Dibromo-2,3,3-trimethyl-3H-indole (1) Geometric parameters (Å, º)

**Table:**

Br1—C3	1.893 (8)	C7—C8	1.494 (13)
Br2—C5	1.894 (9)	C7—C9	1.560 (12)
N1—C1	1.419 (11)	C7—C10	1.531 (13)
N1—C8	1.284 (10)	C8—C11	1.514 (12)
C1—C2	1.381 (11)	C9—H9A	0.9600
C1—C6	1.411 (11)	C9—H9B	0.9600
C2—H2	0.9300	C9—H9C	0.9600
C2—C3	1.374 (11)	C10—H10A	0.9600
C3—C4	1.375 (11)	C10—H10B	0.9600
C4—H4	0.9300	C10—H10C	0.9600
C4—C5	1.412 (11)	C11—H11A	0.9600
C5—C6	1.367 (12)	C11—H11B	0.9600
C6—C7	1.522 (11)	C11—H11C	0.9600
			
C8—N1—C1	105.9 (8)	C8—C7—C10	111.5 (8)
C2—C1—N1	126.0 (8)	C10—C7—C9	110.6 (7)
C2—C1—C6	122.1 (8)	N1—C8—C7	116.7 (8)
C6—C1—N1	111.9 (7)	N1—C8—C11	119.8 (9)
C1—C2—H2	121.3	C7—C8—C11	123.4 (8)
C3—C2—C1	117.5 (8)	C7—C9—H9A	109.5
C3—C2—H2	121.3	C7—C9—H9B	109.5
C2—C3—Br1	118.3 (6)	C7—C9—H9C	109.5
C2—C3—C4	122.6 (7)	H9A—C9—H9B	109.5
C4—C3—Br1	119.1 (6)	H9A—C9—H9C	109.5
C3—C4—H4	120.5	H9B—C9—H9C	109.5
C3—C4—C5	119.0 (8)	C7—C10—H10A	109.5
C5—C4—H4	120.5	C7—C10—H10B	109.5
C4—C5—Br2	117.7 (7)	C7—C10—H10C	109.5
C6—C5—Br2	122.2 (6)	H10A—C10—H10B	109.5
C6—C5—C4	120.2 (8)	H10A—C10—H10C	109.5
C1—C6—C7	106.1 (7)	H10B—C10—H10C	109.5
C5—C6—C1	118.7 (7)	C8—C11—H11A	109.5
C5—C6—C7	135.2 (8)	C8—C11—H11B	109.5
C6—C7—C9	111.5 (8)	C8—C11—H11C	109.5
C6—C7—C10	112.3 (8)	H11A—C11—H11B	109.5
C8—C7—C6	99.3 (7)	H11A—C11—H11C	109.5
C8—C7—C9	111.2 (8)	H11B—C11—H11C	109.5
			
Br1—C3—C4—C5	−180.0 (6)	C3—C4—C5—Br2	−178.1 (6)
Br2—C5—C6—C1	178.3 (6)	C3—C4—C5—C6	1.4 (13)
Br2—C5—C6—C7	−1.3 (14)	C4—C5—C6—C1	−1.2 (12)
N1—C1—C2—C3	−179.1 (7)	C4—C5—C6—C7	179.2 (9)
N1—C1—C6—C5	179.6 (7)	C5—C6—C7—C8	179.9 (9)
N1—C1—C6—C7	−0.6 (9)	C5—C6—C7—C9	62.6 (13)
C1—N1—C8—C7	−0.7 (10)	C5—C6—C7—C10	−62.2 (13)
C1—N1—C8—C11	178.8 (8)	C6—C1—C2—C3	−0.3 (12)
C1—C2—C3—Br1	179.5 (6)	C6—C7—C8—N1	0.3 (9)
C1—C2—C3—C4	0.5 (12)	C6—C7—C8—C11	−179.2 (8)
C1—C6—C7—C8	0.2 (8)	C8—N1—C1—C2	179.8 (8)
C1—C6—C7—C9	−117.1 (8)	C8—N1—C1—C6	0.8 (9)
C1—C6—C7—C10	118.1 (9)	C9—C7—C8—N1	117.9 (8)
C2—C1—C6—C5	0.7 (12)	C9—C7—C8—C11	−61.7 (11)
C2—C1—C6—C7	−179.6 (7)	C10—C7—C8—N1	−118.2 (9)
C2—C3—C4—C5	−1.0 (13)	C10—C7—C8—C11	62.3 (11)

### 4,6-Dibromo-2,3,3-trimethyl-3H-indole (1) Hydrogen-bond geometry (Å, º)

**Table:**

*D*—H···*A*	*D*—H	H···*A*	*D*···*A*	*D*—H···*A*
C3—Br1···N1^i^	1.89	3.19	5.283 (1)	166
C3—Br1···C8^i^	1.89	3.53	5.046 (1)	153
C11—H11*C*···N1^ii^	0.96	2.69	3.621 (1)	164

Symmetry codes: (i) −*x*+1/2, −*y*+1, *z*+1/2; (ii) *x*+1/2, −*y*+3/2, −*z*+1.

### 4,6-Dibromo-2,3,3-trimethyl-3H-indol-1-ium iodide (2) Crystal data

**Table:**

C_12_H_14_Br_2_N^+^·I^−^	*F*(000) = 432
*M**_r_* = 458.96	*D*_x_ = 2.115 Mg m^−^^3^
Monoclinic, *P*2_1_/*m*	Mo *K*α radiation, λ = 0.71073 Å
*a* = 8.3507 (6) Å	Cell parameters from 1781 reflections
*b* = 7.3719 (5) Å	θ = 3.8–28.6°
*c* = 11.7180 (8) Å	µ = 7.74 mm^−^^1^
β = 92.755 (6)°	*T* = 293 K
*V* = 720.53 (9) Å^3^	Needle, red
*Z* = 2	0.4 × 0.2 × 0.1 mm

### 4,6-Dibromo-2,3,3-trimethyl-3H-indol-1-ium iodide (2) Data collection

**Table:**

Xcalibur, Sapphire3 diffractometer	1374 independent reflections
Radiation source: fine-focus sealed X-ray tube, Enhance (Mo) X-ray Source	1242 reflections with *I* > 2σ(*I*)
Graphite monochromator	*R*_int_ = 0.083
Detector resolution: 16.1827 pixels mm^-1^	θ_max_ = 25.0°, θ_min_ = 2.9°
ω scans	*h* = −9→9
Absorption correction: multi-scan (CrysAlisPro; Rigaku OD, 2018)	*k* = −8→8
*T*_min_ = 0.355, *T*_max_ = 1.000	*l* = −12→13
4499 measured reflections	

### 4,6-Dibromo-2,3,3-trimethyl-3H-indol-1-ium iodide (2) Refinement

**Table:**

Refinement on *F*^2^	Primary atom site location: dual
Least-squares matrix: full	Hydrogen site location: inferred from neighbouring sites
*R*[*F*^2^ > 2σ(*F*^2^)] = 0.046	H-atom parameters constrained
*wR*(*F*^2^) = 0.121	*w* = 1/[σ^2^(*F*_o_^2^) + (0.0732*P*)^2^ + 0.1443*P*] where *P* = (*F*_o_^2^ + 2*F*_c_^2^)/3
*S* = 1.05	(Δ/σ)_max_ = 0.001
1374 reflections	Δρ_max_ = 0.86 e Å^−^^3^
97 parameters	Δρ_min_ = −0.91 e Å^−^^3^
0 restraints	

### 4,6-Dibromo-2,3,3-trimethyl-3H-indol-1-ium iodide (2) Special details

**Table:**

Geometry. All esds (except the esd in the dihedral angle between two l.s. planes) are estimated using the full covariance matrix. The cell esds are taken into account individually in the estimation of esds in distances, angles and torsion angles; correlations between esds in cell parameters are only used when they are defined by crystal symmetry. An approximate (isotropic) treatment of cell esds is used for estimating esds involving l.s. planes.

### 4,6-Dibromo-2,3,3-trimethyl-3H-indol-1-ium iodide (2) Fractional atomic coordinates and isotropic or equivalent isotropic displacement parameters (Å^2^)

**Table:**

	*x*	*y*	*z*	*U*_iso_*/*U*_eq_	Occ. (<1)
I1	0.70649 (6)	0.750000	0.12215 (4)	0.0481 (3)	
Br1	0.57546 (9)	0.250000	0.65282 (7)	0.0477 (3)	
Br2	1.19756 (8)	0.250000	0.49523 (7)	0.0455 (3)	
N1	0.7083 (7)	0.250000	0.2129 (5)	0.0333 (13)	
C1	0.7464 (7)	0.250000	0.3305 (5)	0.0310 (15)	
C2	0.6429 (8)	0.250000	0.4171 (6)	0.0339 (15)	
H2	0.532393	0.250000	0.402939	0.041*	
C3	0.7113 (9)	0.250000	0.5275 (6)	0.0361 (16)	
C4	0.8733 (8)	0.250000	0.5472 (6)	0.0334 (15)	
H4	0.915491	0.250000	0.622086	0.040*	
C5	0.9763 (8)	0.250000	0.4585 (6)	0.0321 (14)	
C6	0.9130 (8)	0.250000	0.3468 (6)	0.0317 (15)	
C7	0.9823 (9)	0.250000	0.2291 (6)	0.0328 (15)	
C8	0.8337 (9)	0.250000	0.1513 (6)	0.0347 (16)	
C9	1.0830 (6)	0.4194 (8)	0.2084 (5)	0.0446 (12)	
H9A	1.017880	0.525689	0.215897	0.067*	
H9B	1.122463	0.414922	0.132876	0.067*	
H9C	1.171753	0.423837	0.263544	0.067*	
C10	0.8300 (11)	0.250000	0.0265 (7)	0.0483 (19)	
H10A	0.929629	0.203613	0.001073	0.073*	0.5
H10B	0.814457	0.371640	−0.001140	0.073*	0.5
H10C	0.743463	0.174747	−0.002504	0.073*	0.5
C11	0.5431 (10)	0.250000	0.1659 (8)	0.051 (2)	
H11A	0.541464	0.284350	0.086856	0.077*	0.5
H11B	0.480906	0.334906	0.207372	0.077*	0.5
H11C	0.498349	0.130744	0.172659	0.077*	0.5

### 4,6-Dibromo-2,3,3-trimethyl-3H-indol-1-ium iodide (2) Atomic displacement parameters (Å^2^)

**Table:**

	*U*^11^	*U*^22^	*U*^33^	*U*^12^	*U*^13^	*U*^23^
I1	0.0529 (4)	0.0457 (4)	0.0464 (4)	0.000	0.0092 (3)	0.000
Br1	0.0410 (5)	0.0658 (6)	0.0376 (5)	0.000	0.0156 (4)	0.000
Br2	0.0282 (5)	0.0547 (5)	0.0532 (5)	0.000	−0.0025 (4)	0.000
N1	0.034 (3)	0.038 (3)	0.028 (3)	0.000	−0.006 (2)	0.000
C1	0.024 (4)	0.042 (4)	0.027 (3)	0.000	−0.001 (3)	0.000
C2	0.028 (3)	0.041 (4)	0.033 (4)	0.000	0.004 (3)	0.000
C3	0.036 (4)	0.041 (4)	0.033 (4)	0.000	0.009 (3)	0.000
C4	0.038 (4)	0.038 (4)	0.024 (3)	0.000	−0.004 (3)	0.000
C5	0.031 (4)	0.034 (3)	0.032 (3)	0.000	0.001 (3)	0.000
C6	0.029 (4)	0.033 (4)	0.033 (4)	0.000	0.005 (3)	0.000
C7	0.038 (4)	0.031 (3)	0.030 (4)	0.000	0.014 (3)	0.000
C8	0.040 (4)	0.035 (4)	0.030 (4)	0.000	0.007 (3)	0.000
C9	0.046 (3)	0.047 (3)	0.042 (3)	−0.002 (2)	0.012 (2)	0.007 (2)
C10	0.058 (5)	0.052 (5)	0.036 (4)	0.000	0.006 (4)	0.000
C11	0.038 (5)	0.070 (6)	0.045 (5)	0.000	0.000 (4)	0.000

### 4,6-Dibromo-2,3,3-trimethyl-3H-indol-1-ium iodide (2) Geometric parameters (Å, º)

**Table:**

Br1—C3	1.899 (7)	C7—C8	1.504 (10)
Br2—C5	1.877 (7)	C7—C9	1.532 (7)
N1—C1	1.399 (8)	C7—C9^i^	1.532 (7)
N1—C8	1.301 (9)	C8—C10	1.461 (10)
N1—C11	1.460 (10)	C9—H9A	0.9600
C1—C2	1.364 (10)	C9—H9B	0.9600
C1—C6	1.395 (9)	C9—H9C	0.9600
C2—H2	0.9300	C10—H10A	0.9600
C2—C3	1.389 (10)	C10—H10B	0.9600
C3—C4	1.361 (10)	C10—H10C	0.9600
C4—H4	0.9300	C11—H11A	0.9600
C4—C5	1.381 (10)	C11—H11B	0.9600
C5—C6	1.387 (10)	C11—H11C	0.9600
C6—C7	1.522 (9)		
			
C1—N1—C11	122.5 (6)	C8—C7—C9	110.3 (4)
C8—N1—C1	113.3 (6)	C8—C7—C9^i^	110.3 (4)
C8—N1—C11	124.2 (7)	C9—C7—C9^i^	109.3 (6)
C2—C1—N1	127.6 (6)	N1—C8—C7	109.0 (6)
C2—C1—C6	124.1 (6)	N1—C8—C10	125.2 (7)
C6—C1—N1	108.3 (6)	C10—C8—C7	125.7 (6)
C1—C2—H2	121.7	C7—C9—H9A	109.5
C1—C2—C3	116.5 (6)	C7—C9—H9B	109.5
C3—C2—H2	121.7	C7—C9—H9C	109.5
C2—C3—Br1	119.1 (5)	H9A—C9—H9B	109.5
C4—C3—Br1	119.6 (5)	H9A—C9—H9C	109.5
C4—C3—C2	121.3 (6)	H9B—C9—H9C	109.5
C3—C4—H4	119.3	C8—C10—H10A	109.5
C3—C4—C5	121.5 (6)	C8—C10—H10B	109.5
C5—C4—H4	119.3	C8—C10—H10C	109.5
C4—C5—Br2	118.0 (5)	H10A—C10—H10B	109.5
C4—C5—C6	119.1 (6)	H10A—C10—H10C	109.5
C6—C5—Br2	122.9 (5)	H10B—C10—H10C	109.5
C1—C6—C7	107.2 (6)	N1—C11—H11A	109.5
C5—C6—C1	117.5 (6)	N1—C11—H11B	109.5
C5—C6—C7	135.3 (6)	N1—C11—H11C	109.5
C6—C7—C9^i^	112.3 (4)	H11A—C11—H11B	109.5
C6—C7—C9	112.3 (4)	H11A—C11—H11C	109.5
C8—C7—C6	102.2 (5)	H11B—C11—H11C	109.5
			
Br1—C3—C4—C5	180.000 (2)	C4—C5—C6—C1	0.000 (1)
Br2—C5—C6—C1	180.000 (1)	C4—C5—C6—C7	180.000 (1)
Br2—C5—C6—C7	0.000 (2)	C5—C6—C7—C8	180.000 (1)
N1—C1—C2—C3	180.000 (1)	C5—C6—C7—C9	−61.8 (4)
N1—C1—C6—C5	180.000 (1)	C5—C6—C7—C9^i^	61.8 (4)
N1—C1—C6—C7	0.000 (1)	C6—C1—C2—C3	0.000 (1)
C1—N1—C8—C7	0.000 (1)	C6—C7—C8—N1	0.000 (1)
C1—N1—C8—C10	180.000 (1)	C6—C7—C8—C10	180.000 (1)
C1—C2—C3—Br1	180.000 (1)	C8—N1—C1—C2	180.000 (1)
C1—C2—C3—C4	0.000 (2)	C8—N1—C1—C6	0.000 (1)
C1—C6—C7—C8	0.000 (1)	C9^i^—C7—C8—N1	119.6 (4)
C1—C6—C7—C9^i^	−118.2 (4)	C9—C7—C8—N1	−119.6 (4)
C1—C6—C7—C9	118.2 (4)	C9^i^—C7—C8—C10	−60.4 (4)
C2—C1—C6—C5	0.000 (1)	C9—C7—C8—C10	60.4 (4)
C2—C1—C6—C7	180.000 (1)	C11—N1—C1—C2	0.000 (1)
C2—C3—C4—C5	0.000 (2)	C11—N1—C1—C6	180.000 (1)
C3—C4—C5—Br2	180.000 (1)	C11—N1—C8—C7	180.000 (1)
C3—C4—C5—C6	0.000 (2)	C11—N1—C8—C10	0.000 (1)

Symmetry code: (i) *x*, −*y*+1/2, *z*.

### 4,6-Dibromo-2,3,3-trimethyl-3H-indol-1-ium iodide (2) Hydrogen-bond geometry (Å, º)

**Table:**

*D*—H···*A*	*D*—H	H···*A*	*D*···*A*	*D*—H···*A*
C2—H2···Br2^ii^	0.93	3.05	3.872 (1)	149
C5—Br2···Br1^iii^	1.88 (1)	3.58	5.397 (1)	162
C3—Br1···I1^iv^	1.90 (1)	3.62	5.514 (1)	176
C11—H11*A*···I1^v^	0.96	3.14	3.881 (1)	135

Symmetry codes: (ii) *x*−1, *y*, *z*; (iii) *x*+1, *y*, *z*; (iv) −*x*+1, −*y*+1, −*z*+1; (v) −*x*+1, −*y*+1, −*z*.
